# Data on technopreneurial intention among male and female university students: A comparison

**DOI:** 10.1016/j.dib.2020.106423

**Published:** 2020-10-20

**Authors:** Wei-Loon Koe

**Affiliations:** Universiti Teknologi MARA, Cawangan Melaka, Malaysia

**Keywords:** Comparison, Intention, Post-COVID19, Students, Technopreneurship, University

## Abstract

The data presented were used to examine the level of technopreneurial intention and differences in intention between male and female university students. A total of 361 final-year students from a public university in Malaysia have participated in the data collection process through answering online questionnaire. The respondents were selected by using proportionate stratified sampling. Quantitative method was employed in data analysis process. Specifically, the software of IBM SPSS was used to perform descriptive analysis and independent sample *t*-test. Data have also been tested for internal consistency to ensure their reliability. Mean and standard deviation values for male and female student's technopreneurial intention were presented. In comparison, the data demonstrated that no difference existed between male and female student's technopreneurial intention. The data provided new information on university student's level of technopreneurial intention. It was useful for higher learning institutions and governmental agencies in designing technopreneurship development programs, particularly in the era of post-COVID19 pandemic which requires extensive use of technology in entrepreneurship.

## Specifications Table

**Table:** 

Subject	Business, Management and Accounting (General)
Specific subject area	Entrepreneurship
Type of data	Figure and tables
How data were acquired	Survey questionnaire
Data format	Raw and analysed
Parameters for data collection	Data were collected from a sample of Malaysian final-year undergraduate students selected from a public university. The sample was selected by using stratified sampling method.
Description of data collection	Data were obtained through questionnaire survey. Respondents were required to provide information on their sex and technopreneurial intention. Questionnaire were distributed to respondents via emails and social media platforms.
Data source location	Institution: Universiti Teknologi MARA City/Town/Region: Klang Valley Country: Malaysia
Data accessibility	With the article

## Value of the Data

• Students are considered potential entrepreneurs. Specifically, final-year university students will either become job seekers or job creators very soon. These data are important because they compared the intention to embark on a new breed of entrepreneurship, known as technopreneurship between male and female final-year university students.
• The data provided information on differences in technopreneurial intention exhibited by male and female final-year university students. These data could be useful for higher learning institutions in designing technopreneurship curriculum and technopreneurship development programs to nurture competitive technopreneurs in future.
• The data could be further analysed to describe the extent of technopreneurial intention among final-year university students. They could be applied in other higher learning institutions. They could also be used for further researching the relationship between student's sex and technopreneurial intention.
• The data could provide new information to various governmental agencies in developing technopreneurship among youths. Specifically, they could help the relevant agencies to have a better understanding of differences in technopreneurial intention between male and female university students.
• The data demonstrated final-year university students’ intention to embark on technopreneurship. The development of future technopreneurs is important especially during the post-COVID19 era which requires new normal practices and extensive use of technology such as the Internet, Web applications and automation in entrepreneurship.

## 1 Data Description

The raw data (MS Excel sheet) and questionnaire (MS Word file) are available in the Supplementary Materials section of this article. The data were collected through questionnaire survey from a sample of 361 final-year undergraduate students registered in a public university in Malaysia. The population comprised of 5030 final-year students from three main campuses located in Klang Valley, Malaysia. By referring to Krejcie and Morgan's Sample Determination Table [1], the minimum desired sample size was 357. The sample was selected by using proportionate stratified sampling method. First, the population was stratified into three clusters according to location of campus. Then, sample was selected from each cluster according to its proportion. A total of 450 questionnaires were distributed; however, 380 were returned. After the initial data screening, the usable number of questionnaires was 361. Therefore, the response rate was 80.222%.

The data were quantitative in nature. It was designed to obtain the extent of technopreneurial intention among university students. As university students will leave the formal education system soon and start looking for jobs [2]; thus, it is important to understand technopreneurial intention among them. Due to COVID-19 pandemic, getting employed could be a difficult task for many fresh graduates. Furthermore, IR 4.0 also emphasizes on extensive use of technology. As such, technopreneurship could be a viable career choice for potential university graduates. Intention could be deemed as a predictor of actual behaviour which shows the degree of effort that people exert in a behavior [3]. Therefore, technopreneurial intention was referred as the extent of how hard people trying to embark on technopreneurship. As for the comparison of technopreneurial intention between male and female students, the data were analysed by using independent sample t-test.

**Fig. 1:**
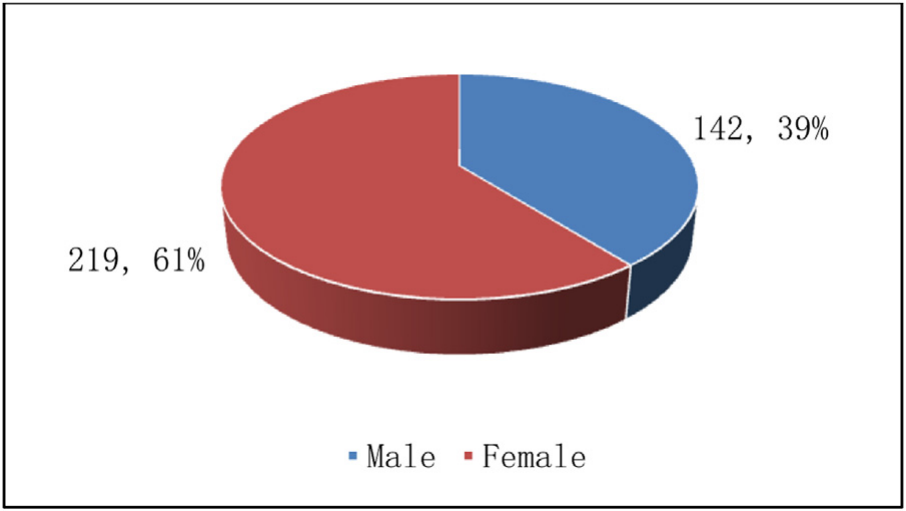
Distribution of respondents by sex

Fig. 1 depicts the distribution of respondents by sex. There were more female students (f = 219; 60.665%) than male students (f = 142; 39.335%). It is worth mentioning that the data were collected from students studied in various programs offered by different clusters of studies, such as business and management, science and technology, and social sciences and humanities.

Since the data were collected through questionnaire, it is important to ensure the reliability of the items. Table 1 illustrates the analysis of internal consistency, in which the Cronbach's alpha (α) value obtained was 0.959. No item was deleted to increase the α value. Therefore, the items were deemed reliable.

**Table 1:** Internal consistency analysis of items.

No of Items	Cronbach's Alpha
6	0.959

Table 2 shows the mean and standard deviation values of individual items of technopreneurial intention by sex. Both male and female students rated highest on item TI 1: Ready to do everything, with *m* = 4.732 (*s* = 1.129) and *m* = 4.808 (*s* = 1.341) respectively. Meanwhile, item TI 2: Professional goal obtained the lowest mean value among male students (*m* = 4.472; *s* = 1.183) and also female students (*m*= 4.585; *s* = 1.432). Slight differences were observed in mean values of each item between male and female students; however, such differences were not statistically tested.

**Table 2:** Mean and standard deviation for individual items of technopreneurial intention.

Items	Sex	Mean	Std. Deviation
TI 1: Ready to do anything	Male	4.732	1.129
	Female	4.808	1.341
TI 2: Professional goal	Male	4.472	1.183
	Female	4.585	1.432
TI 3: Make every effort	Male	4.563	1.139
	Female	4.694	1.372
TI 4: Am determined	Male	4.676	1.224
	Female	4.776	1.381
TI 5: Have very serious thought	Male	4.648	1.300
	Female	4.685	1.419
TI 6: Have firm intention	Male	4.592	1.233
	Female	4.753	1.454

Table 3 demonstrates the statistical analysis for differences in technopreneurial intention for each individual item. To compare the mean values between male and female students, independent sample t-test was performed. Based on the outcomes from Levene's test for equality of variances, item TI 2: Professional goal and item TI 3: Make every effort were significant (Sig.<0.05), while the other items were insignificant (Sig.>0.05). For items which were significant (sig.<0.05) for Levene's test, equal variances were not assume, and vice versa [4]. As a comparison of means for the individual items of technopreneurial intention between male and female students, *t*-test indicated that all items were found to be insignificant. The insignificant outcomes indicated that male and female students did not show differences in individual items of technopreneurial intention.

**Table 3:** Independent sample t-test for individual items of technopreneurial intention.

Items	Levene's Test for equality of variances				
	F	Sig.	t	df	Sig. (2-tailed)
TI 1: Ready to do anything	3.541	0.061	−0.558	359.000	0.577
TI 2: Professional goal	6.011	0.015	−0.812	338.548	0.417
TI 3: Make every effort	4.935	0.027	−0.981	337.778	0.327
TI 4: Am determined	2.050	0.153	−0.704	359.000	0.482
TI 5: Have very serious thought	1.459	0.228	−0.250	359.000	0.803
TI 6: Have firm intention	2.933	0.088	−1.096	359.000	0.274

Table 4 illustrates the outcomes obtained from descriptive analysis for the variable of technopreneurial intention. Male student's technopreneurial intention (*m* = 4.614; *s* = 1.087) was slightly lower than the female student's technopreneurial intention (*m* = 4.717; *s* = 1.279). However, the difference between mean was yet to be proven statistically.

**Table 4:** Mean and standard deviation for the variable of technopreneurial intention.

Variable	Sex	Mean	Std. Deviation
Technopreneurial Intention	Male	4.614	1.087
	Female	4.717	1.279

Table 5 summarizes the independent sample t-test for the variable of technopreneurial intention between male and female students. Levene's test for equality of variances was insignificant; therefore, equal variance was assumed [4]. The insignificant value of *t*-test denoted that there was no difference found in the technopreneurial intention between male and female students.

**Table 5:** Independent sample t-test for the variable of technopreneurial intention.

Variable	Levene's Test for equality of variances				
	F	Sig.	t	df	Sig. (2-tailed)
Technopreneurial Intention	3.723	0.054	−0.792	359	0.429

## 2 Experimental Design, Materials and Methods

Data were collected through a quantitative method which is survey. Quantitative method was deemed appropriate because the variable was measurable [5]. Specifically, self-administered questionnaire was used as the instrument for data collection. Self-administered questionnaire was deemed appropriate because the respondents were university students who were able to read and write without any interference from the researchers. Items for measuring technopreneurial intention were adapted from Liñán and Chen [6]. Since the data was relevant to technopreneurial intention, only items pertaining to entrepreneurial intention were adapted and items measuring other dimensions such as personal attitude, subjective norm and perceived behavioural control were disregarded because they were not relevant to the required data. It is also worth mentioning that the instrument has also been adapted by other researchers in studying technopreneurial intention [7] and social entrepreneurial intention [8]. Adapting items from previous study was to ensure its validity and reliability. Slight modification on the items was performed to suit the context of technopreneurship. The items used seven-point Likert-like rating scale, ranging from 1 = strongly disagree to 7 = strongly agree.

To acquire the desired data, 361 final-year university students who studied in three campuses of a public university in Malaysia were surveyed. The students were selected by using stratified sampling method. In particular, the questionnaire was distributed to the students via email and various social media platforms with the help from Student Representative Council and lecturers. The time frame designed for the data collection was cross-sectional. Two reminders were sent out to ensure the students completed the questionnaire on time.

The software of IBM Statistical Package for Social Sciences (SPSS) was used in the data analysis process. Specifically, independent sample t-test was performed to analyse the data collected. It was deemed appropriate because it compared two means which obtained from two different entities with different conditions [9]. Specifically, in this dataset, comparisons of means between male's and female's technopreneurial intention were performed.

## Ethics Statement

The data was collected from a non-experimental and voluntary survey. However, informed consent was obtained from respondents. The questionnaire was completely anonymous and the respondent's privacy rights was strictly observed.

## Declaration of Competing Interest

The author declares that he has no known competing financial interests or personal relationships which have, or could be perceived to have, influenced the work reported in this article.

## Appendix Supplementary materials

Image, application 1

Image, application 2
