# Supplementary material for: Data on technopreneurial intention among male and female university students: A comparison
Source: Data Brief. 2020 Oct 20;33:106423. doi: 10.1016/j.dib.2020.106423 (PMC7572506; doi:10.1016/j.dib.2020.106423)
Supplement: Supplementary file 1 [file mmc1.docx]

**SURVEY QUESTIONNAIRE**

Aim: This questionnaire aims to collect data regarding student’s technopreneurial intention.

Definition: Technopreneurial intention is defined as “the extent of how hard people trying to embark on technopreneurship”.

Instruction: Please choose ONE description which BEST describes you.

1. What is your gender? (please tick)

Male [ ]

Female [ ]

2. The items below describe your technopreneurial intention. Please circle the degree of your intention from the scale provided below.

1 = Strongly disagree

2 = Disagree

3 = Slightly disagree

4 = Neutral

5 = Slightly agree

6 = Agree

7 = Strongly agree

| i. | I am ready to do anything to be a technopreneur. | 1 | 2 | 3 | 4 | 5 | 6 | 7 |
| --- | --- | --- | --- | --- | --- | --- | --- | --- |
| ii. | My professional goal is to become a technopreneur. | 1 | 2 | 3 | 4 | 5 | 6 | 7 |
| iii. | I will make every effort to start and run my own technology-based firm. | 1 | 2 | 3 | 4 | 5 | 6 | 7 |
| iv. | I am determined to create a technology-based firm in the future. | 1 | 2 | 3 | 4 | 5 | 6 | 7 |
| v. | I have very seriously thought of starting a technology-based firm. | 1 | 2 | 3 | 4 | 5 | 6 | 7 |
| Vi | I have the firm intention to start a technology-based firm someday. | 1 | 2 | 3 | 4 | 5 | 6 | 7 |

= Thank you for your participation =

= The data will be used for research purposes only and remain strictly confidential =
